# Factors Influencing Universal Coverage of AI-Assisted Cervical Cancer Screening: Qualitative Study Based on the Macro Model of Health System

**DOI:** 10.2196/75372

**Published:** 2026-04-01

**Authors:** Lu Ji, Xinke Zhou, Lan Yao

**Affiliations:** 1 School of Nursing, Tongji Medical College, Huazhong University of Science and Technology, Wuhan, China; 2 School of Medicine and Health Management, Tongji Medical College, Huazhong University of Science and Technology, 13 HangKong Road, Wuhan, 430030, China, 86 02783692727

**Keywords:** cervical cancer screening, screening, qualitative research, health systems, artificial intelligence, AI

## Abstract

**Background:**

Improving screening coverage is a central goal of the global strategy to eliminate cervical cancer. In resource-constrained settings, insufficient service accessibility remains a key barrier to expanding coverage. Supported by artificial intelligence (AI)–assisted diagnostic technology, Hubei province has pioneered China’s first provincial-level population-wide cervical cancer screening program, serving 12.67 million eligible women. This initiative provides an innovative practice for addressing such challenges.

**Objective:**

This study systematically examines major factors influencing the achievement of universal screening coverage targets through interviews with core managers and implementers of Hubei province’s screening program. It aims to provide empirical evidence and strategic recommendations for applying AI technologies in cervical cancer screening and enhancing screening coverage rates.

**Methods:**

The interview guide was developed under the guidance of the macro model of health system. A combination of purposive sampling and multistage stratified sampling was used to capture provincial-level overviews and understand regional implementation variations, respectively. Guided by the macro model of health system, interview outlines were developed. Semistructured interviews were conducted between January and August 2024 with key project personnel (one per institution) from 14 relevant institutions. Interview data were analyzed using thematic analysis, with systematic coding and management facilitated by the NVivo software.

**Results:**

Key informants reported that comprehensive screening has been largely achieved. The analysis identified government stewardship, AI-assisted screening technology, screening funding, and health literacy as the major factors for achieving universal screening coverage. Among these, government leadership and the application of AI-assisted diagnostic technologies provide significant driving factors. Additional factors encompassed structural dimensions, including multisectoral coordination, trained screening technicians, and information systems; process dimensions, such as institutional service delivery capacity, quality control measures, and community mobilization; along with outcome dimensions comprising population coverage, cytology positivity rate, follow-up, and treatment rate.

**Conclusions:**

Achieving large-scale cervical cancer screening requires coordinated efforts across four dimensions: government stewardship, screening technology, screening funding, and health literacy. Government stewardship served as the core driver in advancing population-wide screening coverage. Its mechanisms included coordinated procurement of AI-assisted screening services, secured financial investment, formulation of targeted policies, promotion of multi-sectoral collaboration, and optimization of service delivery models. These efforts systematically improved the accessibility and utilization of screening services, ultimately encouraging and facilitating active participation among residents.

## Introduction

Cervical cancer remains one of the major health threats for women worldwide. In 2022, there were approximately 660,000 new cases globally, accounting for 3.3% of total cancer cases, with a mortality rate as high as 56% [1]. Data from the China Cancer Registry Annual Report 2022 show a cervical cancer incidence rate of 13.8 per 100,000 women, with approximately 150,000 new cases annually [2]. In recent years, both incidence and mortality rates have shown an upward trend [3]. The World Health Organization launched a global strategy in 2020 aiming to eliminate cervical cancer through human papillomavirus vaccination, cervical cancer screening, and treatment [4]. Cervical cancer screening is a key focus of policy interventions in China. The current screening rate is approximately 36.8%; however, China aims to achieve a 70% screening rate by 2030 [5].

Improving screening coverage depends on multiple factors, particularly in resource-limited settings where accessible and affordable screening technologies are critically needed [6,7]. Significant disparities in screening coverage exist across nations and regions [8-11], influenced by a combination of factors including sociocultural norms, economic status, health care policies, health awareness, educational resources, and technological capabilities [12-17]. In low- and middle-income countries (LMICs), women commonly face multiple barriers, including limited health literacy, inadequate access to services, insufficient funding, and scarce medical resources, which in turn constrain the improvement of screening coverage rates [18-21]. Both the World Health Organization and the National Institutes of Health recommend the use of artificial intelligence (AI) technology in resource-limited settings to effectively improve access to screening services.

AI-assisted diagnostic technology offers advantages in accessibility, cost, and diagnostic efficiency. Leveraging these strengths, China has implemented its first provincial-level cervical cancer screening program with universal coverage. The shortage of pathologists makes it difficult to implement traditional cytological screening effectively in primary health care institutions, limiting its capacity to adequately meet the population’s screening needs [22,23]. In Hubei province, the adoption of AI-assisted cervical cancer screening technology achieved a coverage rate of 13.45% within 6 months. Calculated with the same number of pathologists, its efficiency was 87.5 times that of manual slide interpretation [24,25]. The AI-assisted approach showed remarkable diagnostic accuracy and cost-effectiveness, with an average per-person cost of 49 RMB [26-28]. This innovative technology has significantly enhanced screening coverage, particularly in resource-limited settings.

Current research on AI technology predominantly focuses on the diagnostic performance of screening techniques, with a notable scarcity of studies investigating its large-scale implementation and associated influencing factors [29-33]. The macro model of health system (MMHS) applies systems thinking to analyze the interplay and interdependence among various components within a health system, making it well-suited for policy interventions and health system diagnostics. This study developed interview guidelines based on the MMHS theoretical framework and conducted interviews with managers and health care providers involved in Hubei’s cervical cancer screening program. The aim was to identify the factors influencing the achievement of universal coverage screening, thereby providing reference suggestions for the wider application of AI-assisted diagnostic technology. Given the core bottleneck of limited cervical cancer screening services due to insufficient pathologist resources in primary health care institutions, this study proposes the following exploratory hypothesis: the introduction of cost-effective and quality-reliable AI-assisted screening technology is expected to substantially overcome the primary barriers to achieving universal screening coverage, thereby accelerating the attainment of coverage targets. Meanwhile, maintaining an open research stance, we developed a theory-driven interview guide to capture the diverse complexities and obstacles that may arise during the implementation of universal coverage programs.

## Methods

### Overview

This study adopts a qualitative approach following Consolidated Criteria for Reporting Qualitative Research (COREQ), the standardized and widely recognized checklist for qualitative research (Checklist 1), and conducted semistructured interviews with managers and implementers of cervical cancer screening programs from 14 institutions. To systematically explore the factors influencing universal cervical cancer screening, the interview guide was developed based on the MMHS. Thematic analysis was performed following a 6-step analytic framework [34], through which textual data were integrated, categorized, and synthesized to ultimately identify the factors affecting the achievement of universal cervical cancer screening coverage. The researchers’ reflections on the predefined statements are presented in detail in the *Discussion* section.

### Macro Model of Health System

#### Framework

**Figure 1.**
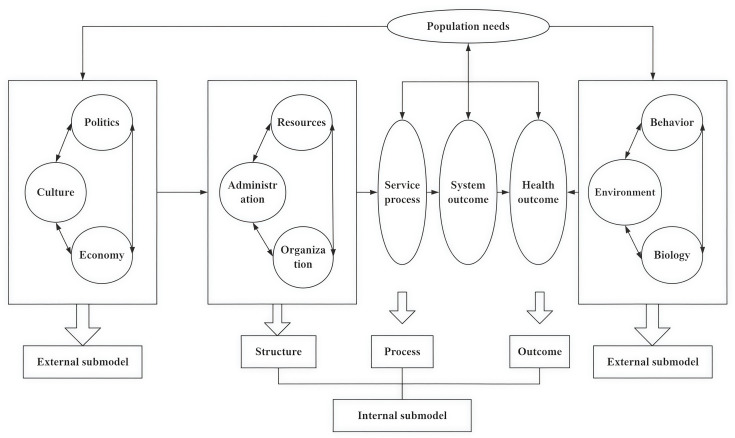
Conceptual framework of the macro model of health system.

The macro model of health system (MMHS) is a theoretical and methodological framework that applies systems thinking to analyze the interactions and interdependencies among the various components within a health system and to reveal its operational patterns [35]. This model integrates systems science, complexity theory, and the theory of social determinants of health to comprehensively explain the combined impact of multiple factors on the health system. The model consists of 2 subsystems: an external submodel and an internal submodel. The external submodel encompasses external factors affecting health, such as politics, economy, culture, population needs, biology, environment, and behavior. The internal submodel represents the internal drivers of health, following the logical framework of structure-process-outcome (Figure 1). Within this framework, administrative, organizational, and resource factors at the structural level influence service delivery processes, which in turn affect system outcomes and ultimately determine health outcomes.

The MMHS is applicable to policy intervention research and health system diagnostics. The comprehensive cervical cancer screening program represents a concrete manifestation of public health policy. The implementation of cervical cancer screening programs is influenced by external factors, such as the policy environment and economic conditions, as well as internal factors including administrative processes, organizational structures, and resource allocation affecting service provision and utilization. These factors ultimately impact screening coverage and population cervical health outcomes. Therefore, this study uses the theoretical framework of the MMHS to design research tools and diagnose the factors influencing the implementation of universal cervical cancer screening.

#### AI-Assisted Cervical Cancer Screening

AI-assisted diagnostic technology has been extensively implemented in China’s free cervical cancer screening programs and has facilitated the completion of the nation’s first provincial-level universal cervical cancer screening project, which provided screening services to approximately 12.67 million eligible women within 3 years. The most notable advantage of this technology lies in its ability to deliver cervical cancer screening of equivalent quality with high efficiency and low cost in resource-limited settings, thereby significantly advancing health equity.

Within the screening workflow, primary health care institutions are only required to collect cervical exfoliated cell samples from participants. The samples are then transported via logistics to laboratories, where they undergo digitization to generate cellular images uploaded to a cloud platform. Diagnosis is performed through a collaborative effort involving AI algorithms, cytotechnicians, and pathologists. Screening participants can quickly access their test results by scanning a QR code [24].

### Study Participants

This study was conducted in Hubei province, China, from January to August 2024. A combination of purposive sampling and multistage stratified sampling was used to obtain an information-rich and representative sample. Hubei province was selected as the study area because it is the first region in China to implement province-wide universal cervical cancer screening.

The sampling procedure was conducted in 2 phases. In the first phase, purposive sampling was applied at the provincial and municipal levels to capture macro-level perspectives on policy-making and program management. Five key administrators were invited from the core management institutions involved in the program. These included 2 officials from the Maternal and Child Health Department of the Provincial/Municipal Health Commission, 2 project managers from the Provincial/Municipal Women’s Federation, and 1 project director from the Provincial Maternal and Child Health Hospital.

**Figure 2.**
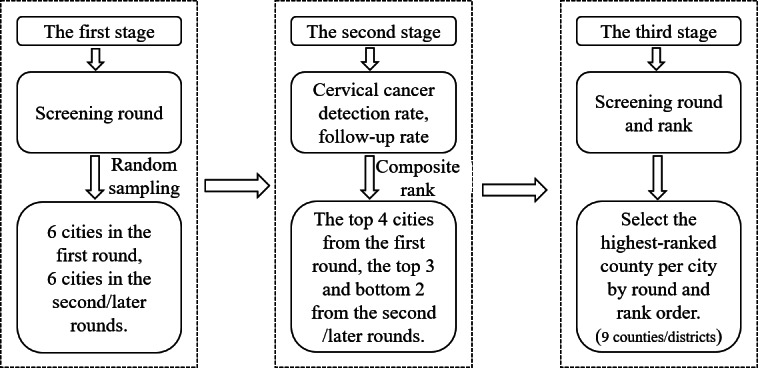
Flowchart of the multistage stratified sampling process.

In the second phase, a multistage stratified sampling method was used to select county-level implementation sites to investigate influencing factors at the grassroots level. The multistage stratified sampling primarily considers factors such as screening rounds, cervical cancer detection rates, and follow-up rates. The detailed sampling flowchart is presented in Figure 2. Based on this framework, 9 county or district-level maternal and child health hospitals were selected as study sites. These hospitals are the sole organizing and implementing institutions for cervical cancer screening within their respective jurisdictions; therefore, their project managers possess comprehensive knowledge of local screening management and operations. One project manager directly responsible for the program was invited from each hospital to participate in an interview. Thus, this study included 14 key informants as interview participants, whose role distribution ensured multilevel perspective coverage from policy formulation to grassroots implementation.

### Data Collection

All participants were informed of the researcher’s institutional affiliation and the purpose of the qualitative study, which was to understand the implementation of the cervical cancer screening program. Following the provision of verbal informed consent by all participants, the data were collected between January and August 2024. All semistructured interviews were conducted by the same researcher (the first author, holding a PhD in management) to ensure consistency in the interview process and interaction style. The interviewer was female, possessed 7 years of qualitative research experience, and had previously conducted specialized interviews related to cervical cancer screening, ensuring a deep understanding of the research context. Of the 14 participants, 2 had prior professional contact with the interviewer from a 2021 project; to mitigate potential bias toward official stances, the interviewer emphasized academic neutrality and proactively probed for challenges, while the remaining 12 were new contacts. Interviews were carried out either face-to-face or by telephone, in private offices or meeting rooms to protect participant confidentiality. Each interview lasted approximately 60 to 80 minutes and was audio-recorded.

### Data Management and Analysis

Within 24 hours post interview, a research team member transcribed the audio recordings verbatim. A second researcher cross-verified transcripts against original recordings to ensure completeness and accuracy. We replaced all personal identifiers with unique codes to anonymize field notes, recordings, and documents. Transcripts were imported into the NVivo 12 (QSR International) software for organization and coding. During the dynamic determination process, we adopted the constant comparative method of “interviewing, coding, and comparing simultaneously.” Two professionals independently completed the transcription and coding of interviews; if there were discrepancies in coding, the experienced interview host made the final decision, which effectively avoided subjective biases caused by different researchers. Based on the inductive thematic analysis method under the MMHS framework, we have analyzed and identified the key influencing factors and have also obtained a comprehensive and systematic set of influencing factors.

Thematic saturation was achieved in this study, with no new themes emerging after the interview with the second participant. In the early stage of the study, the applicability of the MMHS framework was verified through a systematic literature review and expert consultations, and a semi-structured interview guide was developed based on this framework. The guide includes sequential questions targeting influencing factors at different levels, supplemented by keyword prompts to minimize potential information gaps, thereby ensuring the systematicity and completeness of the interview content (Multimedia Appendix 1). Coupled with the subsequent adoption of inductive thematic analysis, which enabled the rapid categorization of data into corresponding thematic categories, the study achieved thematic saturation efficiently.

Furthermore, the findings of the thematic analysis were presented and validated for feedback at a cervical cancer screening seminar with 2 provincial-level key informants, who were also participants in this study. Their perspectives were highly consistent with the study’s findings, and no revision suggestions were proposed.

### Ethical Considerations

Ethics approval was obtained from the Tongji Medical College, Huazhong University of Science and Technology (approval number [2023] IEC(A235)). Prior to the start of each interview—whether conducted face-to-face or via telephone—verbal informed consent was formally obtained from all participants, who were fully informed of the study purpose, procedures, potential risks, and right to withdraw at any time without any negative consequences before their consent was confirmed. All participant data were anonymized and treated in strict confidence. Each participant was offered a financial compensation of 500 RMB (US $70.60) as a recognition of the time they spent (60‐80 minutes per interview) and the professional responses they provided. A portion of participants voluntarily chose to participate on an unpaid basis, aiming to promote the smooth implementation of the full-coverage project to serve women’s health. The compensation was solely intended to offset participants’ time costs and acknowledge their professional inputs, without serving as an incentive to influence the authenticity of their responses.

## Results

### Participant Characteristics

No participants refused or withdrew during the process. Between January and August 2024, we conducted interviews with 14 administrators from the Health Commission (HC), Women’s Federation (WF), and maternal and child health (MCH) hospitals. Participants comprised 9 program managers from MCH hospitals, 2 program directors from provincial HC, and 2 representatives from provincial WF. Among the participants, 92.86% (n=13) reported having more than 10 years of work experience, with 42.86% (n=6) having over 20 years. Most participants held bachelor’s degrees, with 3 participants having completed postgraduate education. Detailed participant characteristics are provided in Table 1.

**Table 1.** Sociodemographic and occupational characteristics of the participants.

Characteristic	Participants, n (%)
Organization	
The Women’s Federation	2 (14.29)
The Maternal and Child Health Hospitals	10 (71.42)
The Health and Wellness Administration	2 (14.29)
Sex	
Male	4 (28.57)
Female	10 (71.42)
Age (y)	
30‐39	2 (14.29)
40‐49	9 (64.29)
50‐59	3 (21.43)
Education	
Associate degree	1 (7.14)
College	10 (71.42)
Master	3 (21.43)
Work experience (y)	
3‐9	1 (7.14)
10‐19	7 (50)
20‐29	6 (42.86)

### Thematic Analysis

#### Overview

Thematic analysis revealed that the core drivers can be summarized into 4 key factors: government stewardship serves as the fundamental guarantee, screening technology provides the foundational support, screening funding supplies sustained momentum, and improving public health literacy is the intrinsic key to achieving broad participation. Further systematic analysis indicates that these factors can be integrated into a comprehensive “structure-process-outcome” framework. Structural factors include government stewardship, multisectoral collaboration, funding, information system, screening technology, and trained screening technicians. Process factors include service delivery capacity, community mobilization, health literacy, and quality control. Outcome measures include coverage, cytology positivity rate, and follow-up rate treatment rate. Detailed results are presented in Table 2.

**Table 2.** Themes and Subthemes.

Themes and subthemes	Tertiary theme
Structure	
Administration	Government stewardship
Organization	Multisectoral collaboration
Resources	Funding for screening Information system Screening technology Trained screening technicians
Process	
Service provision and utilization	Service delivery capacity Community mobilization Health literacy
Outcomes	
Screening results	Coverage Cytology positivity rate Follow-up rate Treatment rate

#### Government Stewardship

Participants indicated that strong government promotion was a key factor enabling the project to achieve comprehensive coverage. This governmental driving force was primarily manifested by incorporating the screening program into regional government performance metrics, with local government departments overseeing HC or WF and MHC in completing the screening tasks. Participants consistently emphasized:

*The government’s proactive approach demonstrates the decisive role of political will. Sustained political support has been the key driver for program implementation, and policy endorsement of AI technology was indispensable for scaling up this AI-assisted screening initiative.*

[MCH01,02,03,04,07,08; HC01]

Another participant noted:

*The provincial government prioritized free cervical cancer screening, incorporating it into the ' public service delivery' performance metrics. Screening completion rates became key evaluation criteria for local governments. Without such government mobilization, universal screening coverage would be unattainable.*

[WF01]

Additionally, the government’s leading role is reflected in its support for screening funding and the application of AI-assisted diagnostic technologies.

#### Screening Technology

Participants indicated that AI-assisted cervical cancer screening technology serves as a critical tool for achieving universal coverage. Previously, primary health care institutions struggled to meet demands of residents due to a shortage of pathologists. By facilitating the decentralization of remote pathological resources, AI-assisted diagnostic technology has enhanced service accessibility and addressed screening bottlenecks in areas with limited health care resources. Furthermore, the screening efficiency of AI-assisted diagnostic technology is remarkably high, enabling significant improvements in screening coverage within a short timeframe.

*AI-based image analysis reduces physicians’ workload and ensures high-quality task completion in primary care settings with insufficient pathology personnel. The technology can automatically handle large volumes of repetitive tasks, such as preliminary screening and marking abnormal areas, thereby alleviating physicians’ workload. Under traditional methods, a pathologist could screen a maximum of 100 individuals per day, whereas digitization increases this capacity to nearly 10,000.*

[MCH03]

*The automated system processes cases much faster than manual methods, and the generated reports are highly accurate. Our AI big data cloud diagnosis platform generates up to 50,000 daily diagnoses.*

[MCH07]

*In our region, manual screening previously allowed only 4,000 cases annually, but with AI technology, we can now screen over 73,000 individuals per year.*

[MCH10]

*Some regions face significant resource limitations, particularly in mountainous areas where specialized pathologists are scarce. The introduction of AI-assisted screening technology helps mitigate these disparities. Through cloud-based API calls, primary care institutions can access advanced diagnostic technology, improving the accessibility of cervical cancer screening.*

[MCH01]

#### Screening Funding

Insufficient screening funding, inadequate matching funds, and unclear allocation collectively constitute the primary financial challenges for the screening program. These issues not only hinder smooth program implementation but also strain health care institutions’ financial capacity and compromise service quality. Some screening institutions report that large-scale screening has caused more severe funding shortages than before.

*The per-case subsidy decreased 25.8% (from 66 RMB to 49 RMB) following AI implementation. But we must cover multiple costs including disposable supplies, field visits of personnel and equipment, and follow-up activities, costs that were underestimated or unaccounted for in the budget, making the 49 RMB subsidy insufficient to cover actual costs. We consistently operate at a loss when conducting screenings*.

[MCH02,07,10]

*Healthcare institutions receive less than 40% government funding, relying entirely on self-generated revenue. However, state funding for program operations remains severely inadequate, and trade union funding has been reduced in recent years—we’re expected to do more work with significantly less funding.*

[MCH04]

Furthermore, insufficient matching funds remain an issue.

*We face tremendous financial pressure. The government assigned heavy screening targets without allocating sufficient funds, requiring advance payments that overextend local government finances*.

[MCH05,08]

Unclear fund allocation exacerbates the funding shortage, remaining a key barrier to achieving universal screening coverage.

*The core funding issue stems from policy-mandated cost estimates set unrealistically low, resulting in systemic underfunding.*

[HC01]

*Current lump-sum allocations lack itemized funding for dual cancers (cervical or breast), with no specific indicators for individual budget lines…*

[MCH07]

*Lump-sum funding becomes problematic at county-level redistribution, with no clear documentation on allocation plans or expenditure purposes.*

[MCH01]

#### Health Literacy

Participants noted that insufficient health literacy among residents is a significant barrier, particularly in rural areas. Although AI-assisted diagnostic technologies for cervical cancer screening have substantially improved service accessibility, residents’ willingness to utilize free screening services remains low. Officials from MCH hospitals emphasized that promoting cervical cancer prevention awareness, disseminating information about free screening, and mobilizing residents to participate require dedicated and proactive efforts, rather than merely providing information about free screening availability.

*If individuals receive invitations, they may participate; however, without direct contact, they might overlook group notifications.*

[MCH08]

*Many are constrained by cognitive limitations and traditional cultural beliefs, while others decline participation due to factors like travel distance to townships or family responsibilities.*

[MCH09]

*Due to insufficient recognition of its importance, incentives such as transportation assistance and free breast cancer screenings are necessary to achieve screening rates.*

[MCH04]

*Project success fundamentally depends on population health literacy—once literacy improves, residents will proactively seek screening without promotional campaigns.*

[MCH07]

*Some women refuse follow-up after initial negative results, mistakenly viewing normal outcomes as permanent risk exemption—reflecting inadequate screening awareness.*

[MCH01]

#### Multisectoral Collaboration

Participants indicated that a multisectoral collaboration mechanism had been established prior to the implementation of the comprehensive coverage project. During the execution of the project, under government stewardship, the responsibilities of each department were further clarified, detailed, and refined. In the process of implementing the comprehensive coverage plan, the MCH were responsible for providing screening services, and the HC oversaw personnel training, project progress, and quality control, while the WF was tasked with mobilizing residents to actively utilize cervical cancer screening services.

*This project is spearheaded by the Women’s Federation and the Hubei Provincial Health Commission, with joint participation from the Trade Union and the Finance Department. The Women’s Federation is responsible for publicity efforts, while the Health Commission provides staff training for maternal and child health hospitals , oversees project progress, and ensures quality control. The Finance Department provides financial support.*

[HC02; MCH01]

#### Information System

Screening programs require the collection of extensive information, and the AI-assisted diagnostic system’s information platform streamlines the entire process for residents—from initial screening to receiving results. The implementation of this information platform has enhanced the management efficiency of screening programs. However, several challenges persist, including incomplete system development, data collection gaps, data sharing difficulties, and limited usability at grassroots levels. Participants reported:

*The system cannot export positive cases, requiring manual entry for each one—extremely inconvenient.*

[MCH02]

Another user noted:

*The system operation is overly complex. A simplified system design would enhance usability.*

[MCH06]

Additionally, concerns were raised about data completeness:

*The system’s statistical data isn’t entirely complete … trade union and corporate data aren’t reported into this system.*

[MCH01]

#### Trained Screening Technicians

Professional personnel required for cervical cancer screening include screening operators, cytopathologists, and diagnostic pathologists, with the shortage of the latter 2 categories being particularly pronounced. Project managers reported that the introduction of AI-assisted diagnostic technology has addressed the shortage of specialized technical personnel in cytological and pathological diagnosis.

*Current staffing remains suboptimal with inadequate professional qualifications. Nevertheless, AI technology can address challenges in cytology and pathology diagnosis, effectively resolving the difficulties caused by a shortage of specialized personnel.*

[MCH01,07; WF02]

*We conduct annual colposcopy training requiring physicians to pass certification exams. Additionally, the provincial MCH hospital offers specialized three-month training programs to ensure sampling proficiency.*

[MCH05,06; HC02]

#### Service Delivery Capacity

In the comprehensive cervical cancer screening program, MCH’s service capacity is primarily reflected in its capabilities for cervical cancer screening and treatment. The screening program encompasses both urban and rural regions, involving provincial or county MCH and township health centers. District or county-level centers exhibit good screening and treatment capabilities. However, township centers face challenges stemming from professional and resource limitations, resulting in restricted screening and treatment capacities. Notably, AI technology has significantly enhanced their screening capabilities.

*Regarding service capacity, most institutions can adequately manage general gynecological diseases and precancerous lesions.*

[MCH02,04,07]

*Township facilities are experiencing downsizing with insufficient obstetrician and gynecologist, limiting maternal and child health follow-up capacity. However, AI-enabled sample referral to provincial centers now ensures successful rural screening implementation.*

[MCH03; HC01]

#### Quality Control

Quality control is essential for ensuring both progress and quality in achieving universal cervical cancer screening coverage. It involves technical accuracy and reliability at the operational level. In addition to on-site inspections, remote monitoring is conducted through the information system, primarily to track completion rates across all screening sites.

*We monitor progress through the information system and reported data, including the number of individuals screened, treated, and managed. These metrics ultimately generate performance rankings.*

[MCH01,03,05]

#### Community Mobilization

Community mobilization campaigns, particularly those driven by administrative initiatives and free voucher distribution, have proven effective in enhancing awareness and increasing cervical cancer screening participation rates. Large-scale community mobilization campaigns can improve public understanding of cervical cancer, thereby increasing screening willingness and advancing the goal of universal screening coverage. These strategies have significantly contributed to achieving comprehensive screening coverage.

*The free cervical cancer screening program will be promoted through flyers, short videos, official social media accounts, television programs, small-scale lectures, and live-streaming sessions.*

[MCH04,05,09]

*Free screening vouchers were printed and distributed to village clinics and women’s committee directors, serving both promotional and participatory purposes. Last year, 100,000 vouchers were printed and distributed based on demand.*

[MCH02]

#### Coverage and Cytology Positivity Rate

Nearly all regions reported achieving comprehensive coverage of cervical cancer screening, primarily attributed to the enhanced service accessibility and efficiency facilitated by AI-assisted technology. Compared to previous methods, the technological upgrades in screening have also significantly increased the cytological positive detection rate. This has further heightened health awareness and willingness to proactively utilize screening services. Coverage and positive detection rates not only directly affect early diagnosis and treatment rates in the current screening cycle but also dynamically shape whether the goal of sustained universal coverage can be achieved.

*The adoption of AI assistance has significantly improved overall detection rates compared to previous methods.*

[MCH01]

With advancements in screening technology and updated equipment, the positive detection rate for cervical cancer screening has increased markedly compared to historical levels. Multiple respondents confirmed:

*Universal coverage has essentially been achieved.*

[MCH02-10]

#### Follow-Up and Treatment Rates

Cervical cancer screening should be conducted at regular intervals to detect lesions at an early stage and enable timely intervention. Participants emphasized that achieving comprehensive screening coverage requires attention not only to the screening rate but, more importantly, to follow-up management and treatment rates. Follow-up and treatment rates are key indicators for the closed-loop management of screening programs, directly influencing their effectiveness and affecting whether residents will continue to participate in screening regularly in the future. Although universal screening coverage has been achieved, significant challenges remain in the follow-up of positive cases, which may, to some extent, affect future screening rates.

*Our follow-up reaches approximately 70% of targets, as many patients don’t respond to phone calls … with higher loss-to-follow-up rates in rural areas.*

[MCH05,10]

Primary challenges include patient noncompliance and disparities between screening, re-screening, and treatment locations. These difficulties complicate treatment rate documentation.

*Some migrant workers relocate before results are available, while many screen-positive patients seek treatment at top-tier hospitals elsewhere—making data tracking challenging though we maintain documentation efforts.*

[WF01,02]

## Discussion

### Principal Findings

Through interviews, we have identified factors influencing the achievement of universal coverage in cervical cancer screening, which span 3 dimensions: structural, service-process, and outcome-related. Among these, government stewardship is considered the primary determinant, followed by the application of AI-assisted diagnostic technology, sufficient funding, and public health literacy, all of which jointly contribute to the realization of comprehensive screening coverage. During implementation, government-led initiatives provide strong support for the promotion of AI-assisted diagnostic technology, multisectoral collaboration, and funding allocation, while also facilitating public access to screening services. Concurrently, AI-assisted diagnostic technology helps alleviate shortages of trained screening technicians, establishes information management platforms, reduces screening costs, and thereby enhances institutional service capacity. To further advance the goal of universal coverage, it is essential to ensure sustained financial investment, improve public health literacy, and strengthen follow-up and treatment for positive cases.

Government stewardship serves as the critical enabler for implementing large-scale cervical cancer screening programs. In developed nations such as the United States, New Zealand, and the United Kingdom, cervical cancer prevention systems are legally mandated, whereas China adopts national policy-driven approaches [36]. Hubei’s universal screening program, integrated into the provincial “Public Service Initiatives,” ties screening targets to local government performance metrics, ensuring comprehensive policy support and fiscal allocation [37]. The program established a multisector framework led by the government, coordinated by the Women’s Federation, with participation from health care, financial, and labor unions. It is noteworthy that even when funding management and public health literacy failed to exert a significant positive influence, the government effectively achieved the phased objective of “testing all who should be tested” through the impetus of clearly defined assessment targets. In contrast, most LMICs lack both legal frameworks and substantive policy support for cervical cancer prevention [38]. Screening in LMICs remains opportunistic, lacking systematic implementation strategies. Thus, robust governmental commitment and policy infrastructure are prerequisites for population-wide screening.

AI-assisted screening technology represents a pivotal component in achieving large-scale cervical cancer screening. It substantially enhances screening efficiency and coverage while reducing costs, enabling Hubei province to complete population-wide screening within compressed timelines. In 2016, a single Chinese pathologist could manually process approximately 100 cytology screenings daily—a rate that would leave millions of women lifetime-unserved by screening programs [39]. However, AI technology allows a single pathologist to conduct roughly 10,000 screenings per day, significantly cutting down mass screening time. The technology also improves accessibility, allowing rural women to undergo sample collection at local clinics without travel burdens. Economically, AI-assisted screening demonstrates superior cost-effectiveness, reducing per-capita costs from 100-200 RMB (medical service fees) to 49 RMB, enabling broader eligibility for free screening. These advantages make universal screening coverage a feasible public health goal.

Financially, the government has allocated approximately 600 million RMB to advance the universal cervical cancer screening initiative. However, it is noteworthy that screening institutions continue to face challenges in funding security, manifested through 3 dimensions: insufficient total funding, lack of matching funds, and ambiguous allocation mechanisms. Funding shortages are particularly acute in LMICs, where systemic underinvestment persists [40,41]. Despite AI’s cost-saving potential, funding gaps endure due to insufficient government reimbursement and overreliance on singular revenue streams [42,43]. Sustainable implementation requires diversified financing mechanisms and evidence-based fiscal policies [44]. Current tiered matching-fund systems fail to alleviate local funding deficits, as reported by stakeholders. This mismatch disproportionately affects economically disadvantaged regions, where heavy reliance on central transfers, limited fiscal autonomy, and low economic capacity exacerbate funding inadequacies [45]. Solutions include needs-based allocation models incorporating regional disparities in fiscal capacity, population size, and geographic coverage. Long-term economic development remains critical for fiscal resilience [46]. China’s lump-sum public health funding—which includes screening budgets—lacks transparent suballocation guidelines [47]. Ambiguities persist regarding cost coverage and interagency fund distribution. Unclear allocation risks resource concentration, compromising efficiency and equity [48]. Standardized operational guidelines for fund distribution are thus imperative for program sustainability [49].

Population health literacy has emerged as a critical determinant of overall health status, reflecting growing public health awareness. It directly influences both health service utilization and treatment-seeking behaviors [50]. This study identified multidimensional barriers to screening participation: low awareness, embarrassment, geographic accessibility issues, and competing household priorities—necessitating incentives, such as gifts or transportation. Knowledge gaps about cervical cancer persist universally across income settings [51]. Despite understanding triennial screening recommendations, women often discontinue after negative results, reflecting insufficient risk perception. Targeted education interventions demonstrably improve screening knowledge and participation rates [52]. Sustainable screening expansion thus requires dual strategies: technological or service optimization and literacy-focused health promotion. Multimodal education can foster proactive health behaviors and sustained screening engagement [53,54].

In the initial phase of our research, we viewed AI technology as the primary driver for achieving universal cervical cancer screening coverage. We believed that addressing the supply bottleneck caused by a shortage of pathologists through technological means would enable rapid attainment of coverage targets. However, through systematic interview analysis, we discovered that while AI technology can effectively enhance service delivery capacity, its effectiveness is highly dependent on systemic support. Government leadership emerged as the most critical factor in achieving screening coverage. By integrating screening into government performance evaluation systems, introducing new technologies (AI-assisted diagnosis), and providing sustained financial support, the government effectively coordinated resources. It mobilized multiple departments—including WFs and MCH hospitals—to collaborate on outreach and organizational efforts, significantly boosting residents’ willingness to participate and actual screening rates. This reflection prompted a fundamental shift in our cognitive framework: moving from an initial “technology-centric” singular assumption to recognizing a multilayered, systematic advancement logic characterized by “government leadership, technological support, multistakeholder collaboration, and competency enhancement.” This shift lends our research conclusions greater explanatory power and policy relevance.

### Strengths and Limitations

Improving cervical cancer screening coverage is a critical public health priority in China and other LMICs. The emergence of AI technology offers a promising solution to enhance the accessibility of these services. Within the context of AI applications in China’s basic public health services, this study systematically investigates the challenges faced in its practical implementation for increasing screening coverage. Furthermore, by applying the MMHS, we provide a comprehensive analysis of the determinants of screening, which offers a robust framework for developing the interview guide. However, a limitation of this study is that it only considers the influencing factors from the perspectives of project managers and providers. In actual program implementation, the perspective of the demand side is equally crucial, and its absence may result in factors affecting program execution being identified incompletely.

### Conclusions

The achievement of comprehensive cervical cancer screening coverage is mainly influenced by government stewardship, AI-assisted screening, screening funding, and health literacy. Among these, government stewardship serves as the core driving force, specifically reflected in the inclusion of screening in government performance evaluations, which drives policy implementation, technological adoption, funding security, multisectoral collaboration, and activities aimed at improving residents’ health literacy and screening willingness. The application of AI-assisted diagnostic technology effectively mitigates constraints, such as shortages of trained screening technicians, limited funding, and insufficient service delivery capacity. To ensure the smooth implementation of the project and achieve preventive goals, coordinated efforts are required in 3 key areas: at the policy level, clear screening plans and supportive measures should be formulated; at the financial level, sufficient funding must be guaranteed, with coordinated multilevel and multidepartmental resources and clearly defined allocation mechanisms; at the service delivery level, diversified health education should be carried out to enhance residents’ health literacy, cultivate regular screening habits, and promote the application of AI technology.

## Supplementary material

10.2196/75372 Multimedia Appendix 1 Interview guide.

10.2196/75372 Checklist 1 COREQ checklist.

## Abbreviations

AI: artificial intelligence
COREQ: Consolidated Criteria for Reporting Qualitative Research
HC: Health Commission
LMIC: low- and middle-income country
MCH: maternal and child health
MMHS: macro model of health system
WF: Women's Federation
