# Supplementary material for: Factors Influencing Universal Coverage of AI-Assisted Cervical Cancer Screening: Qualitative Study Based on the Macro Model of Health System
Source: J Med Internet Res. 2026 Apr 1;28:e75372. doi: 10.2196/75372 (PMC13043005; doi:10.2196/75372)
Supplement: Multimedia Appendix 1 [file jmir-v28-e75372-s001.docx]

**Head of the Health Administration Department**

1. What are the primary responsibilities of our institution in advancing the comprehensive cervical cancer screening program?

2. (Outcome indicator: Coverage rate) What is the current progress of the comprehensive cervical cancer screening plan? As of the end of 2023, 5.26 million individuals have been screened, achieving a coverage rate of 41%. This year is the final year of the plan's implementation. Can full coverage be achieved as scheduled? What are the main challenges currently faced at the management and operational levels?

3. (Outcome Indicator: Follow-up and Treatment) Have there been significant changes in follow-up management rates and treatment rates before and after the comprehensive coverage plan's implementation? What specific challenges do primary healthcare institutions currently face in follow-up management and treatment? What measures should be taken to maintain high follow-up and treatment rates?

4. To achieve widespread coverage of cervical cancer screening services, what key supporting conditions are required at the management, institutional implementation, and community participation levels? In terms of technical support, are there specific requirements for information systems and screening technologies? (Please inquire further about the service provider's capabilities and willingness, management mechanisms, and residents' health knowledge and behaviors.)

5. (Organization/Administration) Which key departments are involved in the comprehensive cervical cancer screening program? What are their respective core responsibilities? What role does our department play in this program, and what are its key priorities? During departmental collaboration, what experiences are worth promoting? What difficulties or challenges have been encountered? Compared to pre-comprehensive coverage efforts, what specific changes have occurred in screening work regarding departmental collaboration?

6. (Funding) Funding Sources and Composition: What are the specific sources and composition of the 600 million yuan screening budget? What are the proportions and amounts allocated from national funds versus provincial and lower-level matching funds? Are there differences in funding allocation standards for different screening populations?

Fund Management and Disbursement: How are project funds allocated, approved, and monitored? What criteria determine the specific allocation amounts when disbursing funds to implementing agencies at various levels? What prominent issues currently exist in project fund management?
Budget Calculation and Allocation: Is the province's total budget of 600 million yuan calculated based on the standard of approximately 12.67 million registered/permanent residents multiplied by 49 yuan per case? Can grassroots healthcare institutions receive full and timely funding allocations and subsidies based on their actual screening numbers?

Cost Breakdown and Funding Support: Within the current 49 yuan per case screening cost breakdown, does the 11 yuan per case allocated to the initial sampling phase at primary screening institutions already include costs required for subsequent follow-up work? Or is there separate funding support specifically designated for follow-up management?

7. (Personnel Training) How is the training program for the “Full Coverage” project designed (training organization, frequency, primary formats (lectures/hands-on practice), core content, assessment methods, etc.)? Since the screening program's launch in 2009, grassroots personnel have received ongoing training. Currently, what specific service capabilities require the most improvement in their actual screening work? Does the “Full Coverage” project's personnel training enhance these capabilities? If improvements in certain areas remain limited, what are the primary reasons?

8. (Quality Control) What forms and practices of quality control have been implemented in project management to ensure overall quality? Using primary healthcare institutions and third-party testing companies as examples, please describe specifically how quality control is conducted. Evaluate the current quality control outcomes: Have the pre-set quality control objectives been achieved? Were these objectives formulated and implemented in accordance with the “Quality Control Manual for Cervical and Breast Cancer Screening”?

9. We are the first province nationwide to implement a comprehensive cervical cancer screening program, a key initiative under the Cervical Cancer Elimination Action Plan. In your view, what factors currently affect the achievement of full coverage in cervical cancer screening?

**Head of the Provincial Maternal and Child Health Project Office**
1. What are the primary responsibilities of our institution in advancing the comprehensive cervical

cancer screening program?

2. (Outcome indicator: Coverage rate) What is the current progress of the comprehensive cervical cancer screening plan? As of the end of 2023, 5.26 million individuals have been screened, achieving a coverage rate of 41%. This year is the final year of the plan's implementation. Can full coverage be achieved as scheduled? What are the main challenges currently faced at the management and operational levels?

3. (Outcome Indicator: Follow-up and Treatment) Have there been significant changes in follow-up management rates and treatment rates before and after the comprehensive coverage plan's implementation? What specific challenges do primary healthcare institutions currently face in follow-up management and treatment? What measures should be taken to maintain high follow-up and treatment rates?

4. To achieve widespread coverage of cervical cancer screening services, what key supporting conditions are required at the management, institutional implementation, and community participation levels? In terms of technical support, are there specific requirements for information systems and screening technologies? (Please inquire further about the service provider's capabilities and willingness, management mechanisms, and residents' health knowledge and behaviors.)

5. (Organization/Administration) Which key departments are involved in the comprehensive cervical cancer screening program? What are their respective core responsibilities? What role does our department play in this program, and what are its key priorities? During departmental collaboration, what experiences are worth promoting? What difficulties or challenges have been encountered? Compared to pre-comprehensive coverage efforts, what specific changes have occurred in screening work regarding departmental collaboration?

6. (Administrative Autonomy: Designation of Primary Screening Institutions and Screening Organization Models) Designation Criteria and Competent Authorities: What are the specific designation criteria (e.g., qualification thresholds, equipment requirements) for primary screening institutions within this county/district? Which institution or department is responsible for final designation?

Number and Distribution of Institutions: Within a county/district, is there only one institution responsible for initial screening tasks, or are there multiple?

Organizational Form Decision-Making: Who determines the organizational form for screening activities within the region (e.g., centralized sites, door-to-door visits)? What are the primary factors considered during decision-making?

7. (Funding) Funding Sources and Composition: What are the specific sources and composition of the 600 million yuan screening budget? What are the proportions and amounts allocated from national funds versus provincial and lower-level matching funds? Are there differences in funding allocation standards for different screening populations?

Fund Management and Disbursement: How are project funds allocated, approved, and monitored? What criteria determine the specific allocation amounts when disbursing funds to implementing agencies at various levels? What prominent issues currently exist in project fund management?
Budget Calculation and Allocation: Is the province's total budget of 600 million yuan calculated based on the standard of approximately 12.67 million registered/permanent residents multiplied by 49 yuan per case? Can grassroots healthcare institutions receive full and timely funding allocations and subsidies based on their actual screening numbers?

Cost Breakdown and Funding Support: Within the current 49 yuan per case screening cost breakdown, does the 11 yuan per case allocated to the initial sampling phase at primary screening institutions already include costs required for subsequent follow-up work? Or is there separate funding support specifically designated for follow-up management?

8. (Personnel Training) How is the training program for the “Full Coverage” project designed (training organization, frequency, primary formats (lectures/hands-on practice), core content, assessment methods, etc.)? Since the screening program's launch in 2009, grassroots personnel have received ongoing training. Currently, what specific service capabilities require the most improvement in their actual screening work? Does the “Full Coverage” project's personnel training enhance these capabilities? If improvements in certain areas remain limited, what are the primary reasons?

9. (Quality Control) What forms and practices of quality control have been implemented in project management to ensure overall quality? Using primary healthcare institutions and third-party testing companies as examples, please describe specifically how quality control is conducted. Evaluate the current quality control outcomes: Have the pre-set quality control objectives been achieved? Were these objectives formulated and implemented in accordance with the “Quality Control Manual for Cervical and Breast Cancer Screening”?

10. We are the first province nationwide to implement a comprehensive cervical cancer screening program, a key initiative under the Cervical Cancer Elimination Action Plan. In your view, what factors currently affect the achievement of full coverage in cervical cancer screening?

**Head of the Women's Federation Department**
1. What are the primary responsibilities of our institution in advancing the comprehensive cervical

cancer screening program?

2. (Outcome indicator: Coverage rate) What is the current progress of the comprehensive cervical cancer screening plan? As of the end of 2023, 5.26 million individuals have been screened, achieving a coverage rate of 41%. This year is the final year of the plan's implementation. Can full coverage be achieved as scheduled? What are the main challenges currently faced at the management and operational levels?

3. (Organization/Administration) Which key departments are involved in the comprehensive cervical cancer screening program? What are their respective core responsibilities? What role does our department play in this program, and what are its key priorities? During departmental collaboration, what experiences are worth promoting? What difficulties or challenges have been encountered? Compared to pre-comprehensive coverage efforts, what specific changes have occurred in screening work regarding departmental collaboration?

4. (Publicity and Mobilization) Regarding publicity efforts, how are publicity campaigns organized and mobilization conducted? What forms do publicity and mobilization take? What experiences have been gained in current publicity and mobilization work? What challenges remain?

5. (Funding) What is the source/composition of the 600 million yuan funding? (How much is allocated by the national government? How do provinces/regions/municipalities provide matching funds? How are funds allocated for different populations?) How are fund disbursements and audits conducted? (Fund disbursement process (institutions/amounts), fund approval) What issues currently exist in project fund management? What are the proposed solutions?

6. We are the first province nationwide to implement a comprehensive cervical cancer screening program, a key initiative under the Cervical Cancer Elimination Action Plan. In your view, what factors currently affect the achievement of full coverage in cervical cancer screening? (What critical conditions are required at the management, service delivery, and community levels, respectively?)

**County/District Maternal and Child Health Hospital Officials**

1. What are the primary responsibilities of our institution in advancing the comprehensive cervical cancer screening program?

2. Since the province-wide “full coverage” initiative launched in 2022, what has been the overall progress of screening efforts in this county/district? Compared to before the plan's implementation, what are the most significant changes in local screening operations regarding organizational models, coverage scope, and service processes?

3. (Outcome Indicator: Coverage Rate) Can we complete the universal screening program on schedule? If not, what are the primary challenges currently faced?

4. (Outcome Indicators: Follow-up and Treatment) Did the follow-up management rate and treatment rate show significant changes before and after the implementation of the universal coverage plan? What are the current challenges we face in follow-up management and treatment?

5. To achieve widespread coverage of cervical cancer screening services, what key supporting conditions are required at the management, institutional implementation, and community participation levels? In terms of technical support, are there specific requirements for information systems and screening technologies? (Please inquire further about the service provider's capabilities and willingness, management mechanisms, and residents' health knowledge and behaviors.)

6. (Organization/Administration) Which key departments are involved in the comprehensive cervical cancer screening program? What are their respective core responsibilities? What role does our department play in this program, and what are its key priorities? During departmental collaboration, what experiences are worth promoting? What difficulties or challenges have been encountered? Compared to pre-comprehensive coverage efforts, what specific changes have occurred in screening work regarding departmental collaboration?

7. (Funding) Is there a funding shortfall, meaning can the allocated screening funds cover the actual screening costs? (If funding is insufficient: If the universal coverage program continues, is our institution willing to sustain this screening work? What are the motivating and hindering factors involved?)

8. (Funding) How are screening project funds allocated to our institution? Which agencies are involved in the disbursement process? Are funds fully allocated based on the number of screened individuals? What issues currently exist in project fund management? What are the proposed solutions?

9. (Information Systems) What types of data are primarily required to be entered into the current screening program's information system? What are the main differences between the “full coverage” information system currently in use and the national “two cancers” information system? What do you consider to be their respective strengths and weaknesses? To better support large-scale population screening and comprehensive management, in what areas do you believe the current information system needs improvement?

10. (Personnel Training) How is the training program for the “Full Coverage” project designed (training organization, frequency, primary formats (lectures/hands-on practice), core content, assessment methods, etc.)? Since the screening program's launch in 2009, grassroots personnel have received ongoing training. Currently, what specific service capabilities require the most improvement in their actual screening work? Does the “Full Coverage” project's personnel training enhance these capabilities? If improvements in certain areas remain limited, what are the primary reasons?

11. (Publicity and Mobilization) How does your institution organize and conduct publicity efforts related to screening? What primary publicity methods are employed? How do you coordinate with other relevant organizations (e.g., communities, women's federations, other medical institutions) to participate in publicity and mobilization work?

12. (Service Capacity): Do you believe your institution has the capacity to independently undertake all screening work for eligible women in this county/district? Specifically regarding screening, follow-up, and treatment, are current staffing and equipment resources adequate?

Can your facility provide treatment services for common gynecological conditions, cervical precancerous lesions, and invasive cervical cancer? For these three disease categories, which are typically treated in-house, and which are most likely to require referral or patient-initiated external treatment? Are there established referral procedures or protocols, or designated referral institutions? 13. (Screening Willingness) How willing are residents to participate in screening (initial screening)? What factors do you believe influence high/low willingness to participate? For patients with positive screening results, follow-up screening is required. Is this facility the only designated follow-up screening institution within the county/district? How do residents receive their screening results, and within what timeframe are results typically available? Which institutions are generally involved in notifying residents for follow-up screening? What is the willingness of residents to participate in follow-up screening, and what factors influence this follow-up screening？

14. (Quality Control) What forms and practices of quality control have been implemented in project management to ensure overall quality? Using primary healthcare institutions and third-party testing companies as examples, please describe specifically how quality control is conducted. Evaluate the current quality control outcomes: Have the pre-set quality control objectives been achieved? Were these objectives formulated and implemented in accordance with the “Quality Control Manual for Cervical and Breast Cancer Screening”?

15. We are the first province nationwide to implement a comprehensive cervical cancer screening program, a key initiative under the Cervical Cancer Elimination Action Plan. In your view, what factors currently affect the achievement of full coverage in cervical cancer screening?
